# In vivo sedative activity of methanolic extract of *Stericulia villosa* Roxb. leaves

**DOI:** 10.1186/s12906-016-1374-8

**Published:** 2016-10-21

**Authors:** Md. Forhad Hossain, Bashudeb Talukder, Mohammad Nasiruddin Rana, Refat Tasnim, Tanzina Sharmin Nipun, S. M. Naim Uddin, S. M. Moazzem Hossen

**Affiliations:** 1 Department of Pharmacy, Institute of Health Technology, Fouzderhat, Chittagong, Bangladesh; 2 School of Allied Health Sciences and Public Health, Walailak University, Thai Buri, Thailand; 3 State University of Bangladesh, Dhaka, Bangladesh; 4 Department of Pharmacy, University of Chittagong, Chittagong, Bangladesh

**Keywords:** Herbal preparations, Sedative properties, CNS depressant activity, Locomotor activity

## Abstract

**Background:**

This plant is very popular ingredient of local made drinks during hot summer. After drinking this drink people feels fresh, relaxed and can enjoy sound sleep. Present study was aimed to assess the sedative properties of a plant *Sterculia villosa* leaves. Therefore, we tried to find out the methanolic extract from the leaves of *Sterculia villosa* leaves having any sedative activity or not.

**Methods:**

The extract were subjected to various in vivo methods like hole cross test, open field test, elevated plus-maze (EPM) test, thiopental sodium induced sleeping time test. Diazepam was used as the standard drug.

**Results:**

From the study, it is clear that the extract has excellent CNS depressant activity by reducing locomotors activity of mice in every cases of hole cross test, open field test, elevated plus-maze (EPM) test compared to the standard diazepam. In addition, the extract prolong the sleeping time (230 min) with quick onset of action (9 min) in contrast to the standard and control group.

**Conclusions:**

From the present study it can be conclude that the extract posses significant a sedative property that may lead to new drug development and further investigation is necessary to understand the underlying mechanisms and to isolate the active principles.

## Background

The plants which are of medicinal value posses some active chemical constituents like alkaloid, tannin, flavonoid and phenolic compounds that can produce physiologic action on human body and hence proven their importance in the health benefit of individuals and communities. In many countries and for many years many researches were oriented towards plants to understand the medicinal uses. From ancient times, most of drugs were of natural origin and at present approximately 30 % of drugs were obtained from plants directly or indirectly [1].

Medicinal plants like Valerian, Passion flower, Melissa, Hops were used as sleep inducers in the treatment of insomnia around the world. Some plants and flowering parts are also used in insomnia treatment as tranquilizers and sleep aids [2].

*Sterculia villosa* Roxb. (Family: Sterculiaceae) is a deciduous, small to large tree found in tropics and subtropics including Bangladesh and is widely known for its several medicinal properties. The plant is used in the treatment of inflammation, seminal weakness, urinary problems, rheumatism, throat infection and also has anthelmintic, antidiabetic, antimicrobial, membrane stabilization and antithrombotic activity; cooling and aphrodisiac properties. This plant is very popular ingredient of local made drinks during hot summer. After drinking this drink people feels fresh, relaxed and can enjoy sound sleep. Therefore, we tried to find out the methanolic extract from the leaves of *Sterculia villosa* Roxb. leaves having any sedative activity or not as the sedative effect of this plant does not investigated yet. Our present work was conducted to establish the sedative effect of methanolic crude extract of *Sterculia villosa* Roxb. leaves using hole cross test, open field test, elevated plus-maze (EPM) test, thiopental sodium induced sleeping time test in Swiss albino mice for the first time [3].

## Methods

### Plant sample collection

Mature and fresh leaves of *Sterculia villosa* Roxb. were collected in 2015 October from Bandorban, Bangladesh. Then the sample was identified and authenticated. A voucher specimen (Accession number: DP/CU/2015/PS-00812) has also been maintained in the herbarium, department of pharmacy, University of Chittagong. Later, the leaves was washed thoroughly, chopped and air dried for a week. Afterwards, dried in the oven at low temperature and powdered in an electric grinder (Moulinex Blender AK-241, Moulinex, France).

### Preparation of extracts

At first, the leaves powder (approximately 500 gm) was kept in a clean, flat bottom glass container which is pre-filled with one litter of methanol. The opening of the container was sealed with foil paper and kept at room temperature for seven days. The contents of the glass container was shaken and stirred irregularly. After seven days, the mixture was filtered through a cotton plug and followed by Whatmenn filter paper No. 1. The filtrate was concentrated by rotary evaporator (RE 200, Sterling, UK) at 45 °C temperature. The yield of the extracts was 4 –5.5 % W/W [4].

### Animals

Swiss albino mice (25–30 gm) were collected from International Centre for Diarrheal Disease and Research, Bangladesh (ICDDR,B) and housed under standard laboratory conditions (relative humidity 55–65 %, room temperature 23.0 ± 2.0 °C) in a 12 h light dark cycle and acclimatized for 7 days where the experiment would take place. The animals were fed with standard rodent food and water formulated by ICDDR, B. This experiment involving animals was approved by the Ethical Review Committee at the Department of Pharmacy, University of Chittagong, Bangladesh and conducted according to their guidelines.

### Trial registration

For experimental clinical study on animal trial registration and permission was issued from departmental clinical ethical review committee, department of pharmacy, University of Chittagong. The trail registration reference number is ERC/DP/CU/2015/0011.

### Statistical analysis

Results were expressed as mean ± SEM. One-way ANOVA was used for analysis of data. Differences were considered significant at *P* ≤ 0.05.

### Hole cross test

In this experiment, a steel cage having a size of 30 × 20 × 14 cm was used. A partition which has a hole of 3 cm diameter at a height of 7.5 cm was fixed in the middle of the cage. At first, the animals were divided into three groups namely control, positive and test containing five animals in each group. Then, in the test group methanolic extract of *Sterculia villosa* Roxb. at a dose of 400 mg/kg body weight were administered orally and followed by the control and positive control group were treated with vehicle (1 % Tween 80 in water at a dose of 10 ml Kg^-1^ per oral) and diazepam (at a dose of 1 mg Kg^-1^ intra peritoneal) respectively. After that, the animals were introduced into the cage and the number of passages from one chamber to other through the hole inside the cage was counted for 3 min on 0, 30, 60, 90 and 120 min intervals [5].

### Open field test

In this experiment, open fields apparatus consist of 30 cm × 50 cm white box with walls of 27 cm were used to study the spontaneous locomotive and exploratory activity in mice. At first, the area of the open field were divided into a series of square blocks and colored black and white alternatively. The apparatus were kept in a light and sound-attenuated room. Then, the animals were divided into three groups namely test, control and positive control consist of five animals in each group. After that, the animals were treated with leaves extract (400 mg/kg body weight, orally), vehicle (1 % Tween 80 in water at a dose of 10 ml Kg^-1^ per oral) and diazepam (at a dose of 1 mg Kg^-1^ intra peritoneal). The mice were then kept into the apparatus and the number of square blocks visited by each mouse was calculated for 3 min on 0, 30, 60, 90 and 120 min intervals [6].

### Elevated plus-maze (EPM) test

This test was performed according to a previously described method [7] with minor modifications. The maze apparatus was consisting of two open arms (5 × 10 × 0.5 cm) and two closed arms (5 × 10 × l5 cm). The arms were radiated from a central platform (5 × 5 cm) and elevated to a height of 40 cm above the floor. The entire maze apparatus was made of dark opaque wood. The animals were divided into two groups (*n* = 5) and treated with *Stericulia villosa* leaves extract (400 mg/kg body weight, orally) and diazepam (at a dose of 1 mg Kg^-1^ intra peritoneal). After sixty minutes, each animal was placed at the centre of the maze and observed for five minutes. Then, the number of open arm entries which is defining as entry of all four paws into an arm was recorded.

### Thiopental sodium induced sleeping time test

In this test, the effect of *Stericulia villosa* leaves extract on thiopental sodium induced sleeping time on mice was evaluated by a previously described method with a small modification. Swiss albino mice were separated into three groups of five animals each. In the test group leaves extract (400 mg/kg body weight, orally) was given, the control group receive vehicle (1 % Tween 80 in water at a dose of 10 ml Kg^-1^ per oral) and diazepam (at a dose of 1 mg Kg^-1^ intra peritoneal) was administered to the positive control group. Then, twenty minutes later each animals were treated with thiopental sodium (40 mg/kg body weight, intraperitoneally). After that, the animals were observed for the latent period (time between thiopental administrations to loss of righting reflex) and duration of sleep (time between the loss and recovery of righting reflex) [8].

## Results

### Hole cross test

In the evaluation of sedative effects of *Stericulia villosa* we have started our journey with hole cross test and open field test to record the spontaneous locomotor activity. The result was presented on Table 1. From the result it is evident that *Stericulia villosa* caused a marked reduction in the number of hole crossed and such inhibitory effect was started at 30 min and continued up to 120 min after administration of methanolic extract of *Stericulia villosa*.

**Table 1:** CNS depressant activity of methanolic extract of leaves of *Stericulia villosa*

Time (Minute)	Hole cross test			Open field test		
	Control	Diazepam	MESV	Control	Diazepam	MESV
0	18.67 ± 1.08	13.00 ± 1.41	14.67 ± 2.08	135 ± 3.50	105.33 ± 2.08	147.3333 ± 12.34
30	16.67 ± 1.78	6.00 ± 1.87	11.33 ± 2.51	65.67 ± 1.32	59.00 ± 0.76	69.33 ± 16.92
60	14.33 ± 1.08	4.33 ± 0.40	9 ± 1.00	49.33 ± 1.60	34.00 ± 2.29	43 ± 13.11
90	12.67 ± 1.08	3.67 ± 0.81	7.66 ± 0.57	46.33 ± 1.04	19.67 ± 0.76	38.33 ± 6.51
120	11 ± 0.70	1.33 ± 0.40	8.67 ± 1.53	48.33 ± 2.36	17.67 ± 1.15	34.66 ± 5.13

All values are expressed as mean ± SEM (*n* = 5); One way Analysis of Variance (ANOVA). *P < 0.05, significant compared to control

### Open field test

The result was represented in Table 1. From the result it is evident that, the plant produces sedative effect (decrease spontaneous locomotion). The sedative effect was found at 30 min and continued up to 120 min.

### Elevated plus-maze (EPM) test

The results of EPM test were presented in the Table 2. The result revealed that diazepam at a very small dose (1 mg/kg) has significant percentage of open arm entry. Methanol extract of *Stericulia villosa* at a dose of 400 mg/kg also substantially increase the percentage of open arm entry.

**Table 2:** CNS depressant activity of *Stericulia villosa* extract on thiopental sodium induced sleeping time test in mice

Group	Treatment, Dose & Route (p.o)	Thiopental sodium induced sleeping time test		Elevated plus-maze (EPM) test
		Onset of sleep (Min)	Duration of sleep (Min)	% Entry into open arm
Control	1 % tween 80 in water, 10 ml/kg	40 ± 3.701	62 ± 2.121	12.30 ± 2.286*
Standard	Diazepam, 1 mg/kg	11.80 ± 1.9235*	180 ± 7.694*	29.40 ± 3.286*
Test	MESV, 400 mg/kg	9 ± 4.336*	230 ± 5.805*	42 ± 9.970*

All values are expressed as mean ± SEM (*n* = 5); One way Analysis of Variance (ANOVA). *P < 0.05, significant compared to control

### Thiopental sodium induced sleeping time test

The sedative effect of the *Stericulia villosa* leaves extract was confirmed upon pretreatment of the animals with the plant extract lengthen the sleeping time and shorten the sleep latency (Table 2). Effect of *Stericulia villosa* on sleep latency and sleeping time is more intense than diazepam which is a standard sedative drug [9]. *Stericulia villosa* exhibits synergistic sedative and hypnotic action with thiopental sodium.

## Discussion

The drug Diazepam which belongs to the benzodiazepine group is a central nervous system depressant used in the management of sleeps disorders such as insomnia. Benzodiazepines have a binding site on GABA receptor type-ionophore complex [8]. They decrease activity, moderate excitement and calm the recipient. Substances like diazepam (the reference drug used in this study) reduce onset of and increase duration of barbiturate-induced sleep and reduce exploratory activity possessing potentials as sedative [8].

In both test, Hole cross test and Open field test any agents with sedative property will reduce the number of locomotion, understood as of lacking curiosity to new environment [8]. Locomotor activity is an indicator of mental wakefulness or alertness and decrease locomotion which is indicative of calmness and sedation could be interpret as reduced CNS excitability [10]. *Stericulia villosa* leaves extract decrease the number of hole crossed and the number of square blocks crossed in 30 min and subsequently influenced the locomotor activity in mice which indicates the sedative property.

The elevated plus-maze is considered as a popular model of animal anxiety and hence justified for used in rats and mice [7]. The EPM test was performed here to evaluate the anxiolytic potential of *Stericulia villosa*. The parameter which was examined in the EPM test namely percent of open arm entry are sensitive to agents are thought to act through the GABA_A_ receptor complex and validate the use of diazepam as a positive control. Diazepam confirms the anxiolytic effects by increasing the percentage of open arm entry [7]. The results of the EPM test clearly disclose that methanolic extract of *Stericulia villosa* has anxiolytic potential.

Thiopental sodium belongs to the group barbiturate and induces sleep in both humans and rodents. The thiopental sodium induced sleeping time test in mice was used to investigate the sedative – hypnotic drugs [8]. In our study, oral administration of *Stericulia villosa* extract 20 min before the thiopental sodium induces sleep and similar effects were observed with diazepam. Much evidence have suggested that, CNS depressant barbiturates like Thiopental sodium binds to the barbiturate binding site on the GABA receptor complex and stimulate GABA mediated hyper polarization of postsynaptic neurons [8]. From our results close relationship between the sedative effect of *Stericulia villosa* and diazepam could be suggested.

Phytochemical investigation of methanol extract *Stericulia villosa* under this study explored the presence of medicinally active secondary metabolites alkaloids, glycoside, steroids, tannins, terpenoids and flavonoids. The sedative and anxiolytic potential may be due to the presence of those secondary metabolites.

## Conclusion

The findings of our study strongly validate the rapid, long-lasting and statistically significant sedative activity of *Stericulia villosa*. Though, further extensive research is needed to isolate the active principles of the plants and to understand the underlying mechanism behind the pharmacological activity of this plant.
